# Supporting the future of medicine: Student mental health services in medical school

**DOI:** 10.3389/frhs.2023.1032317

**Published:** 2023-03-09

**Authors:** Elijah W. Hale, Rachel A. Davis

**Affiliations:** ^1^ School of Medicine, University of Colorado, Aurora, CO, United Status; ^2^ Department of Psychiatry, University of Colorado, Aurora, CO, United States

**Keywords:** student mental health and wellness, policy, handbook evaluation, student services, mental health support

## Abstract

**Background:**

Mental health issues are common among United States medical students, and the AAMC has established recommendations for student mental health services provided by medical schools. Few studies directly compare mental health services at medical schools across the United States and, to our knowledge, none analyze how well schools adhere to the established AAMC recommendations.

**Objective:**

To determine whether mental health services at United States medical schools adhere to established guidelines.

**Methods:**

From October 2021 to March 2022, we obtained student handbooks and policy manuals from 77% of the accredited LCME United States medical schools. The AAMC guidelines were operationalized and placed into a rubric format. Each set of handbooks was independently scored against this rubric. A total of 120 handbooks were scored and the results were compiled.

**Results:**

Rates of comprehensive adherence were very low, with only 13.3% of schools displaying adherence to the full set of AAMC guidelines. Partial adherence was higher, with 46.7% of schools meeting at least one of three guidelines. Portions of guidelines whose requirements reflected a standard for LCME accreditation displayed a higher rate of adherence.

**Conclusion:**

The low rates of adherence across medical schools, as measured by handbooks and Policies & Procedures manuals, represents an opportunity to improve the mental health services within United States allopathic schools. An increase in adherence could be a step towards improving the mental health of United States medical students.

## Introduction

In recent years, medical schools have sought to foster wellness with encouragement of activities such as mindfulness, exercise, peer connections, healthy diet, and self-care (1). However, mental health is an often overlooked aspect of wellness, despite the higher rates of depression, burnout, and suicidal ideation present in medical students compared to the general population (2, 3). Therefore, it is necessary to ensure students are receiving adequate support and mental health services to best support their patients and themselves. However, there is little research available on the information provided to medical students regarding the availability of mental health services.

Both the Association of American Medical Colleges (AAMC) and the Liaison Committee on Medical Education recognize medical student mental health as a vital aspect of education and suggest the availability of counseling, and the AAMC has a publicly available set of guidelines regarding mental health services for students at United States allopathic schools (Table 1) (4, 5). Despite widespread recognition of the existing barriers to mental health care, the AAMC guidelines remain the only publicly available document guiding the structure of mental health services offered to medical students in the United States. Additionally, the authors are not aware of any research directly comparing the availability of mental health services across United States medical schools. However, the availability of substance use treatment for medical students has been assessed by examination of school handbooks and policy manuals (6). Using similar methodology to evaluate the accessibility of mental health services in medical schools, we reviewed medical school student handbooks and Policies & Procedures (*P* & *P*) manuals to assess adherence to the established AAMC guidelines.

**Table 1:** Operationalized rubric of AAMC mental health guidelines for medical schools.

AAMC Guideline (2) by clause	Indeterminate	Adherent
1a. Schools should provide access to confidential counseling by mental health professionals for all students. Institutional policies regarding the confidentiality of mental health service records for medical students should be established.	Instructions on how to access information on available mental health services.	Mention and existence of the availability of explicitly confidential counseling services for medical students.
1b. These policies should make the necessary distinction between voluntary and administratively mandated evaluation and/or treatment.	Instructions on how to access policies regarding mandatory evaluation/treatment.	Mention of situations and/or criteria leading to mandatory evaluations and/or treatment.
1c. For administratively mandated evaluation, disclosure of evaluation and/or treatment results should be limited to those who required the evaluation and should be in accordance with federal or state laws governing the disclosure of confidential information.	Instructions on how to access further policies regarding confidentiality of evaluations.	Declaration of confidentiality expectations regarding mandated evaluations.
2a. Schools should have guidelines regarding the utilization of mental health professionals and/or records of assessment and treatment by mental health professionals in proceedings regarding student advancement and dismissal.	Instructions on how to access further policies regarding record use in advancement and dismissal.	Mention of use of mental health treatment records and/or assessment records with regards to advancement and dismissal, in line with relevant privacy legislation.
2b. The committee recommends that evaluation and/or treatment of students be undertaken by non-teaching faculty or at a minimum, by different individuals than those rendering advancement or promotion decisions.	Instructions on how to access information on policies on non-involvement by faculty in evaluation and/or treatment.	Explicit statement that evaluation and/or treatment will be performed by non-teaching faculty who are not rendering advancement or promotion decisions.
3. Schools should publish and regularly update a list of available mental health assessment and counseling services, the institutional assurance of confidentiality, the means of access, and the associated costs for their students.	Instructions on how to access a list of resources containing information on the following: available mental healthcare services, confidentiality policies, accessibility, and cost.	Existence of a list of resources containing information on the following: available mental healthcare services, confidentiality policies, accessibility, and cost.

## Methods

From October 2021 to March 2022, we sought to obtain medical student handbooks and Policies & Procedures (*P* & *P*) manuals from the 155 LCME accredited US allopathic medical schools. Most documents were easily accessible *via* the schools' main websites. When a document was not readily available online, we contacted the medical school's office of student affairs or equivalent office *via* email and phone a maximum of three times. Schools replying to contact after the month of March 2022 were not included in the analysis. Documents were examined when they were identified as either a “student handbook” or “policy & procedure manual” through title or by confirmation *via* school representatives.

The AAMC guidelines for mental health services were separated into clauses, and then operationalized into a rubric (Table 1). Each set of documents was scored according to the ternary categorical rubric, with the three categories being “adherent,” “indeterminate,” and “nonadherent.” Guidelines were considered “adherent” when all the rubric criteria were met. The term “indeterminate” was applied to criteria when documents contained instructions on where to obtain the corresponding information, but did not provide the information within the text. This term was chosen as the completeness of the information provided was unclear, and the multiple steps required to access the information presented a potential boundary to care. For example, a document with the statement “Inquire at the Office of Student Affairs for information on mental health services” would be labelled “indeterminate” for criteria 1a, as it does not provide any information on the availability of confidential counseling but does provide an avenue for further inquiry. Documents were considered “nonadherent” to a criteria when the information was not present in the text, and no further information on where to locate the information was presented.

The scoring of documents was performed by EWH, and frequency of criteria adherence was added into a spreadsheet. In order to ensure consistency, results were intermittently spot checked. When all documents had been scored, we generated descriptive statistics based on the frequency count of adherence for each criterion. This structure and publication of this study followed the most recent Standards for Quality Improvement Reporting Excellence (SQUIRE) guidelines (7). The Colorado Multiple Institutional Review Board (COMIRB) designated this study as non-human research and not in need of review.

## Results

Out of 155 schools, we were successful in obtaining documents from 120 (77.4%). Of the outstanding 35, six schools denied access to the documents, and the remaining 29 did not respond to repeated requests. A final 120 (77.4%) sets of school documents were inspected. Document length ranged from 14 to 454 pages.

**Figure 1:**
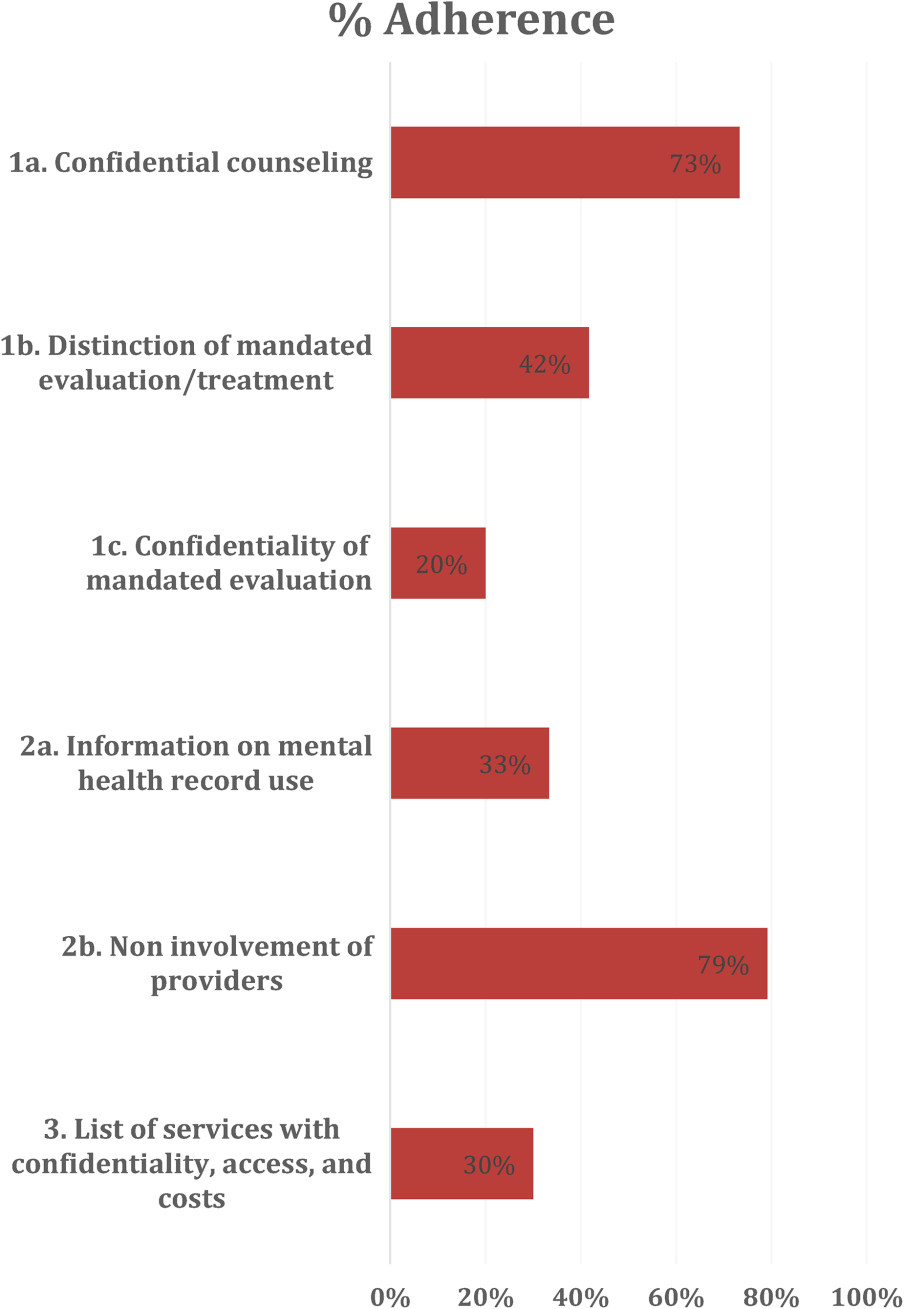
School document adherence to criteria, in percentage of total schools analyzed.

Of the 120 sets of documents analyzed, sixteen (13.3%) adhered to all AAMC guidelines for mental health services. Thirty-three schools (27.5%) fulfilled the criteria for only one AAMC guideline, and sixty-four schools (53.3%) did not fulfill the criteria for any AAMC guideline. Guideline 2 had the highest frequency of adherence, with 39 schools (32.5%) fulfilling criteria. Individual criteria had higher rates of adherence. Criterion 2b, which details non-involvement of psychiatric providers in evaluation of patient-students, was adhered to by 95 schools (79.2%). The second highest criterion, with 88 sets of documents (73.3%) adhering, was 1a, detailing the availability of confidential counseling for students (Table 2). These results are graphically represented in Figure 1.

**Table 2:** Adherence, nonadherence, and indeterminate adherence by AAMC guideline.

AAMC Guideline	Adherent	Nonadherent	Indeterminate
#1 – All criteria met	20 (16.7%)		
#1 – At least one criterion met	73 (60.8%)		
#1 – Zero criteria met		27 (22.5%)	
1a. Confidential counseling with confidential records	88 (73.3%)	22 (18.3%)	10 (8.3%)
1b. Explicit distinction of mandated evaluation/treatment	50 (41.7%)	70 (58.3%)	0 (0%)
1c. Confidentiality of mandated evaluation	24 (20%)	86 (71.7%)	10 (8.3%)
#2 – All criteria met	39 (32.5%)		
#2 – At least one criterion met	60 (50%)		
#2 – Zero criteria met		21 (17.5%)	
2a. Use of mental health professionals/records	40 (33.3%)	75 (62.5%)	5 (4.2%)
2b. Non-involvement of providers	95 (79.2%)	25 (20.8%)	0 (0%)
#3. List of services, confidentiality, access, costs	36 (30%)	19 (15.8%)	65 (54.2%)
**Total # of guidelines met:**	The total # of guidelines met only demonstrates comprehensive, rather than partial, adherence.
No Guidelines		64 (53.3%)
One Guideline		33 (27.5%)
Two Guidelines		7 (5.8%)
All Guidelines		16 (13.3%)

## Discussion

Our findings demonstrate the need for increased focus on provision of mental health treatment. Fewer than 14% of schools fully adhere to all of the established AAMC guidelines. Even one of the most commonly fulfilled criteria, availability of confidential counseling services, was present in fewer than 75% of the published documents. In fact, our data show that most medical school documents did not adhere to any of the AAMC guidelines. The highest degree of adherence was to criterion 2b, which is directly related to the LCME standard of accreditation which requires that health professionals involved in psychiatric or psychological care of a medical student not be involved in that student's assessment or promotion (5). This LCME standard also recommends availability of student counseling, but the recommendation is not compulsory (5). This LCME standard relating to criterion 2b and 1a may contribute to their high rate of adherence. The other criteria are not reflected in LCME standards, and there is a drop in adherence of 29% between criterion 2b and the third most fulfilled criterion, 1b, which details the importance of separating mandatory evaluation and elective treatment. One possibility for the difference could be that medical schools' abidance to the AAMC guidelines is driven by accreditation standards. This presents the possibility that adherence to these guidelines could be improved by adapting the LCME standards to include specific requirements regarding student mental health services. If accreditation standards mandated the availability of student mental health services, it is likely that more schools would offer these services and provide information in their handbooks and official *P* & *P* manuals.

Medical students have unique mental health needs. They face intense and increasing competition in nearly all aspects of their training, from the rising standards of admission to the increasingly competitive residency match (8, 9). As a result, medical students are under immense academic and emotional stress and regularly report perceiving limited support from their medical schools, which may be one of many factors that contribute to their high rates of depression and anxiety (10). In spite of medical training in psychiatric illness, students largely report not seeking treatment for their own mental health needs, even when they recognize those needs as unmet (11). Untreated mental illness puts medical students at risk across a variety of domains. In their personal health, medical students may experience increased substance use or severe infectious illness COVID-19, both of which have demonstrated increased prevalence in individuals with untreated mental illness (12, 13). Within school, medical students with underlying mental illness may experience higher rates of sleep issues and poorer academic performance (14). Even after graduating, they may experience higher rates of burnout and even suicidality (15). It is incumbent upon medical schools to teach students how to mitigate this stress by instilling curriculum supporting the development help-seeking and self-care skills into their professionalism curriculum, as well as offering ample mental health resources for students.

While overall wellness includes mental health, many medical schools provide resources that primarily focus on other aspects such as meditation, exercise, and an increase in coaching relationships (1). While these aspects are important, a focus on wellness in the absence of a similar focus on and normalization of mental health treatment may lead to feelings of inadequacy, shame, or imposter syndrome when students are unable to independently “be well.” (16) Furthermore, it can also increase stigma around trainees who may be considered “unwell,” including those with disabilities (17). In an AAMC report, learners with disabilities described many barriers within their medical education, including lack of clear policies/procedures and lack of access to health care and wellness supports (18). Centralizing information in readily available school documents could help remove lack of resource awareness as a potential barrier to mental health treatment. Additionally, the handbooks offer an opportunity to reduce feelings of imposter syndrome for struggling learners by providing an institutional acknowledgment that the use of resources is encouraged (19).

There are both strengths and limitations inherent to the use of school documents as proxies for availability of mental health services. While it is possible that handbooks and official *P* & *P* manuals may not represent all information presented to students, and therefore underestimate rates of adherence, the handbook is often a central resource for students seeking information. More than 80% of document sets had statements indicating medical students were required to read and understand the information within, which indicates they should be a reliable reflection of school resources. Nonetheless, schools may provide information regarding mental health services through other avenues, such as email, websites, lectures, or campus announcements. Conversely, it is also possible that student handbooks provide a more robust description of services than what is realistically available to students, due to factors such as psychiatric provider shortages. For example, a school may report availability of confidential counseling, but the waitlist for an appointment may exceed several months, greatly hindering student access.

Our investigation provides an examination into the availability of mental health services at United States allopathic medical schools. Our data suggests a lack of adherence to AAMC guidelines, despite the prevalence of mental health issues among medical students and the established importance of mental health treatment. Improving the information contained within student handbooks and *P* & *P* manuals, as well as offering services recommended by the AAMC, could help reduce barriers to mental health treatment. As the awareness of barriers to psychiatric care increases, it becomes even more important that medical students are aware of resources available to support their mental health. Regardless of their chosen specialty, medical students and future physicians will ultimately be relied upon to support patients' mental and emotional health at different points in their training. Adequate mental health services must be provided to ensure students' behavioral health is maintained and supported throughout their medical education.

## Data Availability

The raw data supporting the conclusions of this article will be made available by the authors, without undue reservation.
